# From Dish to Trial: Building Translational Models of ALS

**DOI:** 10.3390/cells15030247

**Published:** 2026-01-27

**Authors:** Ilias Salamotas, Sotiria Stavropoulou De Lorenzo, Aggeliki Stachtiari, Apostolos Taxiarchis, Magda Tsolaki, Iliana Michailidou, Elisavet Preza

**Affiliations:** 1 Laboratory of Neurodegenerative Disease, Center for Interdisciplinary Research and Innovation, Aristotle University of Thessaloniki, 57001 Thessaloniki, Greecetsolakim1@gmail.com (M.T.); elliepreza@gmail.com (E.P.); 2 Department of Molecular Medicine and Surgery, Karolinska Institutet, and Clinical Chemistry, Karolinska University Laboratory, Karolinska University Hospital, 17166 Stockholm, Sweden; apostolos.taxiarchis@regionstockholm.se; 3 Division of Science and Technology, Anatolia American University, Pylaia, 55535 Thessaloniki, Greece

**Keywords:** sporadic ALS, preclinical models, iPSC, therapeutic strategies, clinical trials, organoids, NMJ

## Abstract

**Highlights:**

**What are the main findings?**

- Patient-derived iPSCs enable human-relevant modeling of ALS pathology.
- 3D cultures and ALS-on-a-chip systems improve mechanistic understanding of ALS.

**What are the implications of the main findings?**

- Large-scale sporadic cohorts may support ALS clinical trials.
- Proposed iPSC framework for ALS drug development.

**Abstract:**

Amyotrophic lateral sclerosis (ALS) is the most common motor neuron disease, marked by progressive degeneration of upper and lower motor neurons. Clinically, genetically, and pathologically heterogeneous, ALS poses a major challenge for disease modeling and therapeutic translation. Over the past two decades, induced pluripotent stem cells (iPSCs) have reshaped our understanding of ALS pathogenesis and emerged as a promising translational platform for therapy development. ALS modeling has further expanded with the advent of three-dimensional systems, including ALS-on-chip platforms and organoid models, which better capture cell–cell interactions and tissue-level phenotypes. Despite these advances, effective disease-modifying therapies remain elusive. Recent clinical trial setbacks highlight the need for improved trial design alongside robust, translational iPSC models that can better predict therapeutic response. Nonetheless, the outlook is promising as large iPSC patient cohorts, quantitative phenotyping combined with genetically informed patient stratification, and reverse translational research are beginning to close the gap between in vitro discovery and clinical testing. In this review, we summarize the major advances in iPSC technology and highlight key iPSC-based studies of sporadic ALS. We further discuss emerging examples of iPSC-informed therapeutic strategies and outline the challenges associated with translating iPSC-derived mechanistic insights and pharmacological findings into successful clinical therapies.

## 1. Introduction to Amyotrophic Lateral Sclerosis (ALS)

Amyotrophic lateral sclerosis (ALS) is a progressive neurodegenerative disorder, and the most common form of motor neuron disease (MND) [1]. It is characterized by selective degeneration of upper motor neurons (UMNs) in the motor cortex, and lower motor neurons (LMNs) in the brainstem and spinal cord, leading to progressive muscle weakness, paralysis and ultimately respiratory failure and death [2]. The global prevalence of ALS is estimated at 4–6 cases per 100,000, with regional differences reflecting a combination of genetic, environmental, and diagnostic factors [3]. On average, disease onset is in late midlife and survival ranges from 2 to 5 years after symptom onset [2]. ALS shares clinical features with other MNDs, including progressive muscular atrophy (PMA), progressive bulbar palsy (PBP), and primary lateral sclerosis (PLS), which can complicate diagnostic classification, especially early in the disease course. Importantly, ALS shows substantial clinical, genetic, and neuropathological overlap with frontotemporal dementia (FTD), and the two disorders are now regarded as opposites of an ALS–FTD disease spectrum rather than distinct entities [4].

Approximately 10% of ALS cases are familial (fALS) with at least another affected family member, whereas the remaining 90% are considered sporadic (sALS) [5]. The most common genetic cause of ALS is the GGGGCC (G4C2) repeat expansion in *C9orf72*, which accounts for up to 40% of fALS and 7–10% of sALS [6,7]. Other major ALS genes include *SOD1*, *TARDBP* and *FUS* [8]. The frequencies of several ALS mutations differ between populations of different geographical regions due to founder effects [9]. Recent technological advances in exome and whole-genome sequencing have led to the discovery of additional ALS genes, including *TUBA4A* [10], *MATR3* [11], *CHCHD10* [12], *TBK1* [13], *NEK1* [14], *CCNF* [15] and *KIF5A* [16,17], whose potential pathogenic roles are currently under investigation.

A major neuropathological hallmark of ALS is the nuclear depletion and cytoplasmic aggregation of phosphorylated and ubiquitinated TDP-43 in motor neurons and glia, which is present in the majority of ALS cases [18,19]. Typical TDP-43 pathology is absent from *SOD1* or *FUS* mutation carriers, who instead exhibit cytoplasmic aggregates of SOD1 [20] or FUS proteins [21,22], respectively. *C9orf72* repeat expansion carriers display typical TDP-43 pathology but also exhibit characteristic p62-positive dipeptide repeat protein (DRP) aggregates produced via G4C2 repeat-associated non-ATG (RAN) translation in the cerebellum and hippocampus [4,23].

**Figure 1:**
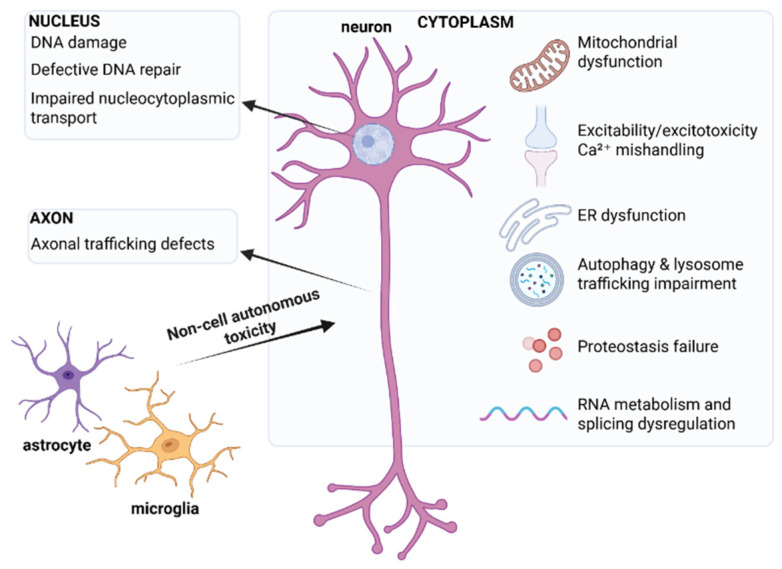
Insights from familial ALS iPSC models into convergent dysregulated pathways across *C9orf72*, *TARDBP*, *SOD1*, and *FUS*. Created in BioRender. Michailidou, I. (2026) https://BioRender.com/r3ncwwb (accessed on 21 January 2026).

From a disease modeling perspective, iPSCs have transformed ALS research and the wider neurodegeneration field by allowing human disease mechanisms to be studied in a robust, patient-specific and physiologically relevant manner. Early ALS iPSC studies established some of the first robust human cell-based disease models, largely focusing on familial ALS and recapitulating hallmark features, including SOD1 aggregation, TDP-43 mislocalization, *C9orf72* sense/antisense RNA foci and dipeptide repeat (DPR) proteins, and cytoplasmic FUS inclusions. Over time, and with increasing evidence of significant involvement of immunity and inflammation in ALS development, models have expanded from cell-autonomous neuronal to complex microfluidic co-culture and organoid systems incorporating diverse cell types, including glia. Despite gene-specific phenotypes, studies of *C9orf72*, *TARDBP*, *SOD1*, and *FUS* converge on shared dysregulated pathways: (i) proteostasis and autophagy failure, (ii) RNA metabolism and splicing defects, (iii) mitochondrial and ER dysfunction, (iv) excitability and Ca^2+^ handling abnormalities, (v) axonal transport and synaptic pathology, (vi) DNA damage and repair defects, and (vii) glial-mediated inflammation and toxicity (Figure 1).

To date, riluzole, edaravone, and the *SOD1*-targeted antisense tofersen are widely approved disease-modifying therapies for ALS [24,25,26]. The first drug approved for ALS in 1995 was Riluzole, a glutamate antagonist that exhibits neuroprotective effects, delays the need for ventilatory support, and extends survival by approximately three months [24]. Edaravone, an antioxidant and free radical scavenger, was approved by the FDA in 2017 [25]. It has been shown to slow disease progression, particularly in early-stage ALS. Finally, tofersen, an intrathecally administered antisense oligonucleotide (ASO) targeting *SOD1* mRNA, received accelerated FDA approval in 2023 for adults with ALS associated with a pathogenic *SOD1* mutation [26]. As demonstrated in the phase 3 VALOR trial, tofersen administration led to a reduction in cerebrospinal fluid (CSF) SOD1 protein and plasma neurofilament light chain (NfL), but no significant difference in the Amyotrophic Lateral Sclerosis Functional Rating Scale–Revised (ALSFRS-R) score, the primary clinical endpoint of the trial. Recent data from longer-term analyses show sustained biomarker effects and suggest that earlier and continuous tofersen treatment may slow functional decline in *SOD1*-ALS [27].

Despite progress in the field, currently available ALS therapies show limited clinical benefit, and *SOD1*-ASO treatment has a very narrow target population, approximately 1–2% of ALS patients. Closing the translational gap will likely require model systems that capture key, patient-relevant disease mechanisms, enable scalable and quantitative assessment of therapeutic effects, and support stratification of heterogeneous patient populations. In this context, human iPSC-based platforms offer a translational route to connect genotype, cellular phenotype, and drug response in ways that can better inform target selection, biomarker development, and clinical trial design.

Here, we review advances in iPSC technology over the two decades since its discovery in the context of ALS and discuss the emerging three-dimensional (3D) iPSC platforms transforming ALS research. We also focus on iPSC studies of sALS, which accounts for 90% of ALS cases, and presents distinct challenges in terms of genetic architecture and phenotypic heterogeneity. Finally, we discuss emerging examples of iPSC-informed therapeutic approaches and outline the challenges in translating iPSC-derived mechanistic and pharmacological findings into successful clinical therapies.

## 2. The Evolution of iPSC Technologies Enabling ALS Modeling

The discovery of somatic reprogramming twenty years ago, in 2006, by Takahashi and Yamanaka [28] provided the foundation for disease modeling using patient-derived cells and marked the beginning of a new era for studying human diseases, including ALS. Reprogramming of human dermal fibroblasts to iPSCs was achieved via overexpression of four transcription factors *OCT-4*, *SOX2*, *KLF4*, and *c-MYC* (OSKM) [29]. This was the crucial first step that not only provided an unlimited source of cells identical to stem cells but also allowed the study of disease mechanisms in a range of differentiated cell types that retain the patient’s genetic background. Importantly, this technology arrived at a time when knowledge about ALS pathogenesis was based on postmortem examination, animal and cell line overexpression experiments, and filled a gap in human-relevant ALS modeling. Since their first description, reprogramming protocols have gradually shifted from the use of integrative DNA vectors towards integration-free methods that preserve genomic integrity and improve the reliability of ALS iPSC phenotypes such as episomal plasmids [30], non-integrating Sendai viral vectors [31,32], synthetic modified mRNA [33], and self-replicating RNA systems [34].

In ALS, adapting this technology for disease modeling required the development and refinement of robust differentiation protocols, particularly for generating ALS-relevant motor neurons and supportive glial cell types. The first ALS iPSC model was described in 2008, establishing the first proof-of-principle that ALS pathology can be studied in patient-derived motor neurons [35]. Since these early milestones, the field of ALS iPSC research has been under continuous expansion and refinement, with the goal of creating faithful and translational ALS models. Early studies focused on mutation-defined fALS iPSC models, exploring cell-autonomous motor neuron phenotypes. To this end, several iPSC-MN differentiation protocols have been described and over time refined for increased efficiency, purity, and reproducibility [36]. These protocols are generally based on the same differentiation principles: an initial neural induction achieved by dual-SMAD inhibition [37], followed by caudalization and ventralization towards a spinal motor neuron fate using retinoic acid (RA) and Sonic hedgehog (SHH) pathway activation, respectively [38], and a final maturation phase. As non-cell-autonomous contributions became widely recognized, astrocytic and microglial models were integrated into ALS iPSC studies [36]. More recently, the emergence of 3D spinal and cortico-motor organoids, neuromuscular junction (NMJ) models, and microfluidic organ-on-chip systems has offered unprecedented fidelity for modeling human motor circuits and multicellular interactions in ALS [39,40,41].

Other advances in genome engineering, multi-electrode arrays (MEAs), microfluidics and artificial intelligence (AI) technologies have fundamentally shaped the field. Genome engineering via Transcription Activator-Like Effector Nucleases (TALENs) [42] and more recently CRISPR/Cas9 [43] allowed precise isogenic controls and dissection of genotype–phenotype relationships within human iPSC models. Notably, TALEN-mediated repair of *SOD1* mutations was the first demonstration that genome repair can rescue ALS-relevant motor neuron phenotypes in isogenic lines [44]. Nowadays, CRISPR/Cas9 nucleases are the gold standard for knockout, knock-in, and correction strategies in ALS iPSC research [45]. MEA technology has had a major impact on functional phenotyping by enabling scalable electrophysiology, leading to the discovery of early motor neuron hyperexcitability across multiple fALS genotypes (*SOD1*, *C9orf72*, *FUS*) [46]. Recent platforms include high-density MEAs (HD MEAs) featuring higher electrode densities [47], and 3D MEAs featuring electrodes that extend into or wrap around 3D organoids [48]. Similarly, microfluidic platforms gradually progressed from soma–axon compartmentalization [49] to synapse-enriched and pre/postsynaptic compartmentalization [50], then to neuron–glia co-cultures allowing the investigation of non-cell-autonomous mechanisms [51], and most recently to complex organ-on-chip platforms that incorporate flow for improved maturation and translational relevance [52]. Finally, the shift towards multicellular 3D models and large sALS iPSC cohorts has generated high-dimensional datasets spanning integrated multi-omics, high-content imaging, electrophysiology, and diverse functional readouts, making machine learning and AI increasingly important for extracting disease-relevant patterns and phenotypic clustering [53,54,55,56].

**Figure 2:**
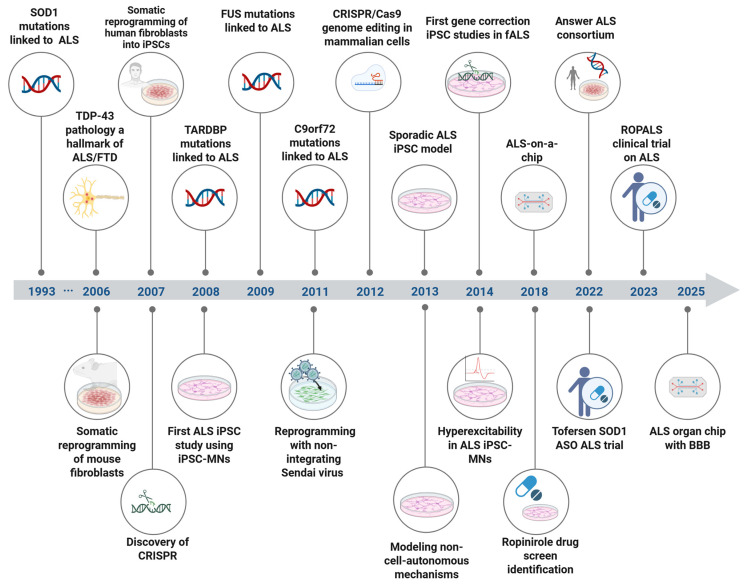
Overview of major milestones in ALS research and advances that enabled iPSC modeling of ALS. Created in BioRender. Michailidou, I. (2026) https://BioRender.com/2rssage (accessed on 21 January 2026).

In conclusion, continuous advances in iPSC modeling, together with the incorporation of emerging technologies, have yielded state-of-the-art experimental platforms for ALS, enabling mechanistic dissection of disease pathways, modeling of patient heterogeneity, and preclinical therapeutic development (Figure 2).

## 3. Emerging 3D Platforms Paving the Way to Faithful ALS Modeling: Focus on the NMJ

Clinical and experimental data strongly suggest that the neuromuscular junction (NMJ) is one of the earliest sites of pathology in ALS, with dying-back denervation and NMJ dismantling preceding excessive motor neuron loss [57,58]. A central limitation of conventional iPSC co-cultures is that they lack the tissue-level architecture and mechanical context needed to model the neuromuscular junction (NMJ) as a functional, multicellular unit. New 3D iPSC platforms are therefore adding further value to existing iPSC ALS models by providing insights into NMJ physiology, via functional readouts of synaptic dysfunction including impaired contraction, altered circuit coupling, or progressive denervation. Here we describe some of the emerging 3D modeling systems, their features and their application in ALS NMJ modeling (Table 1).

Three-dimensional motor-unit chips and other microphysiological systems have had a major impact on ALS modeling as they combine engineered tissue structure with quantitative functional outputs. One of the earliest compartmentalized human “ALS-on-a-chip” motor-unit platforms combines 3D skeletal muscle bundles and iPSC-MN spheroids in a microfluidic device, allowing axons to extend, NMJs to form, and muscle contraction to be quantified as a primary readout of neuromuscular function [39]. The MN spheroids are made optogenetically responsive by expressing channelrhodopsin-2 (ChR2), so presynaptic activity can be triggered with light and directly translated into stimulation-evoked muscle contractions. A sALS motor unit demonstrated the modeling capabilities of this system by recapitulating ALS phenotypes, including reduced neurite outgrowth and NMJ formation, alongside weaker contractile output and increased markers of muscle stress/apoptosis. The platform also accommodated chronic glutamate exposure, which gradually disrupted neurite architecture and further impaired contractile performance. Importantly, this platform can feature an iPSC-derived endothelial cell (iEC) barrier, and supports functional pharmacology, strengthening its translational relevance. As demonstrated, treatment with rapamycin and/or bosutinib improved contraction phenotypes and reduced muscle apoptosis, consistent with engagement of autophagy-related pathways.

Another study described the development of human iPSC-derived sensorimotor organoids, featuring multicellular neuromuscular tissue and functional NMJs in long-term culture [40]. For their generation, neuromesodermal progenitor-like spheres were differentiated under adherent conditions to yield organoids containing motor neurons and skeletal muscle, together with additional cell types including sensory neurons, astrocytes, microglia, and vascular-like populations. The function of the NMJ was quantified using muscle contraction as the primary physiological endpoint. The contractions were triggered by ChR2-optogenetic neuronal activation and blocked by agents such as curare and botulinum toxin, consistent with synaptic transmission-dependent muscle contraction. Importantly, when applied to ALS modeling, organoids derived from sporadic and familial ALS iPSC lines exhibited deficits in NMJ-associated structural and/or functional measures relative to controls. Similar NMJ phenotypes were also observed in isogenic CRISPR/Cas9-edited fALS lines carrying mutations in *TARDBP* (G298S), *SOD1* (G85R), and *PFN1* (G118V) on a common genetic background.

Another important development in the field was the description of human iPSC-derived trunk neuromuscular organoids (NMOs) which are produced by differentiation of iPSCs into neuromesodermal progenitors (NMPs) then into self-organizing 3D NMOs that develop a neural and a muscle compartment [41]. NMOs feature motor neurons, spinal interneurons, skeletal muscle, and importantly terminal Schwann cells. The presence of terminal Schwann cells is emphasized because these non-myelinating glial cells are part of the NMJ, with key roles in NMJ formation, maturation, maintenance and repair, yet commonly missing from many in vitro NMJ/neuromuscular culture models [59]. When applied to *C9orf72* ALS iPSCs to model fALS, the resulting *C9orf72* NMOs displayed peripheral neuromuscular defects consistent with reduced muscle contractions, NMJ denervation, and loss of Schwann cells, as well as *C9orf72* pathological hallmarks of RNA foci and DPRs in the neural compartment. Acute treatment with the unfolded protein response inhibitor GSK2606414 both improved glutamate-evoked muscle contraction and reduced DPR aggregation and autophagy-related changes, demonstrating another insightful paradigm of functional pharmacology using 3D iPSC platforms.

Finally, assembloids are useful models for reconstructing longer-range motor circuitry upstream of the NMJ [60]. Cortico-motor assembloids are generated by fusing cortical spheroids (hCS) with hindbrain/spinal cord spheroids (hSpS) and then incorporating skeletal muscle spheroids (hSkM). Within the assembloids the corticofugal neurons extend projections into the spinal spheroid and form functional connections, while spinal-derived motor neurons engage the muscle module. Impressively, circuit functionality closely resembles human top-down control, whereby stimulating the cortical compartment via glutamate uncaging or optogenetics drives robust muscle contraction, and curare sensitivity supports that this output depends on NMJ signaling rather than spontaneous muscle activity alone. Although CMOs have not yet been widely adopted for ALS iPSC disease modeling, they offer a compelling direction for future work because they can, in principle, support investigation of the full UMN-LMN-NMJ axis within a single human, experimentally accessible system, enabling questions about how dysfunction emerges and propagates across connected nodes of the motor circuit in ALS.

**Table 1:** Emerging 3D modeling systems, their features and their application in ALS NMJ modeling.

	“ALS-on-a-Chip” Motor Unit [39]	Sensorimotor Organoids [40]	Neuromuscular Organoids [41]	Cortico-Motor Assembloids [60]
**Model** **description**	Compartmentalized microfluidic 3D motor-unit chip combining 3D iPSC-derived skeletal muscle bundles (on pillars) with optogenetic (ChR2) MN spheroids to form NMJs, with additional iPSC-derived endothelial cell (iEC) barrier	Adherent neuromesodermal “sensorimotor organoid” cultures containing motor neurons + skeletal muscle + sensory neurons, astrocytes, microglia, vasculature, forming NMJs across many iPSC lines (patient-derived + isogenic fALS edits)	“Trunk” neuromuscular organoids derived from *C9orf72* ALS iPSCs + isogenic controls, comprising spinal cord neural and peripheral muscular tissues (incl. Schwann cells), designed to model spinal/peripheral neuromuscular pathology	Modular fusion of cortical spheroids + hindbrain/cervical spinal cord spheroids + skeletal muscle spheroids to self-assemble a multi-synaptic cortico-spinal–muscle circuit in 3D
**ALS model**	sALS	fALS	*C9orf72* ALS	-
**Functional readouts**	Stimulation-evoked muscle contraction quantified (pillar deflection/force); MN viability and NMJ formation	Motor neuron-dependent muscle contractions (spontaneous and optogenetically evoked); NMJ structural metrics (α-BTX AChR clusters apposed to presynaptic markers; EM confirmation) and innervation/NMJ area quantification	Contractile weakness/reduced contractile frequency, denervated NMJs, and neural activity readouts, including MEA-based assessments	Circuit function measured by muscle contraction triggered by glutamate uncaging or optogenetic stimulation of cortex; calcium imaging, rabies tracing, and patch-clamp to confirm connectivity
**ALS** **phenotype captured**	ALS MNs showed slower neurite outgrowth, reduced NMJ formation, weaker contractions, and increased muscle apoptosis/atrophy signals vs. control motor units	Across ALS patient lines and isogenic fALS edits, organoids showed NMJ impairment, detected by reduced contraction and immunocytochemical NMJ/innervation deficits (e.g., reduced innervated NMJs/NMJ area in specific genotypes)	C9-ALS NMOs recapitulated peripheral ALS-like phenotypes: contraction weakness, neural denervation, loss of Schwann cells, plus C9 hallmarks (RNA foci and DPR proteins) in neurons/astrocytes	Platform development not ALS disease model
**Functional pharmacology**	Rapamycin and bosutinib and co-treatment improved contraction-related deficits and reduced muscle apoptosis	-	Acute GSK2606414 (UPR inhibitor) increased glutamatergic muscular contraction and reduced DPR and autophagy-related readouts in the model	-

## 4. Modeling Sporadic ALS

For many years, mutation-defined fALS iPSC models have been at the center of ALS research, but sALS iPSC models are now increasingly used for capturing the genetic and phenotypic heterogeneity of ALS and for enabling mechanistic studies and therapeutic testing with broader relevance to the wider ALS patient population (Table 2). One of the earliest sALS iPSC modeling studies showed that sALS iPSC-MNs can develop de novo TDP-43 aggregates under basal conditions [61]. Subsequent high-content chemical screening led to the identification of FDA-approved small molecule modulators of TDP-43 aggregation, demonstrating the feasibility of sALS iPSC models for drug discovery [61]. Another study of ALS iPSC models, including sALS lines, has also detected TDP-43 aggregates in sALS iPSC-MNs via electron microscopy, as well as neurofilament (NF) aggregates and reduced mitochondrial density in neurites of both familial and sporadic ALS iPSC-MNs [62]. ALS iPSC-MNs were vulnerable to treatment with the proteasome inhibitor MG132, exhibiting TDP-43 translocation, worsened NF inclusions, mitochondrial distribution impairment, and caspase-3 activation [62]. Additional transcriptomic analyses of sALS iPSC-MNs have revealed dysregulation of gene networks involved in mitochondrial function [63], while MEA recordings have identified intrinsic hyperexcitability in sALS cervical iPSC-MNs [64]. Beyond iPSC-MNs, non-cell-autonomous mechanisms have also been explored by transplanting sALS iPSC-astrocytes into the spinal cord of mice, where they integrate and induce progressive neuronal degeneration in vivo, confirming astrocyte-mediated toxicity in sALS [65]. Another more recent study reported no overt TDP-43 mislocalization or increased apoptosis in sALS iPSC-MNs, but instead progressive axonal pathology consistent with impaired mitochondrial axonal transport, early neurite/axonal outgrowth defects, and reduced capacity to form functional NMJs in co-culture with human primary myoblast-derived myotubes [66]. The severity of these axonal phenotypes correlated with patients’ clinical progression, consistent with previous reports linking neurite outgrowth defects to ALSFRS-R scores [67] and suggests that targeting axonal and NMJ dysfunction may represent a promising therapeutic avenue in sALS [66].

From 2018 onwards, a clear shift towards modeling sALS has been driven largely by a small number of large-cohort studies that offered scale and standardization paradigms in ALS modeling. A particularly important milestone in this direction has been the establishment of the Answer ALS consortium, designed to accelerate ALS treatment discovery by generating large, clinically well-annotated, and standardized human iPSC cohorts for sporadic and familial ALS [68].

An influential study addressed the heterogeneity of sALS by generating iPSCs from 32 sALS patients, differentiating them into motor neuron-enriched cultures, and tracking a longitudinal panel of disease-relevant phenotypes [67]. These included neurite dynamics, LDH cytotoxicity, stress granules, protein mislocalization/aggregation, and cleaved caspase-3 apoptosis. The analysis stratified sALS lines into subgroups, with many showing FUS-like and/or TDP-43-like cellular features rather than a SOD1-like profile, indicating mechanistically distinct sALS subtypes. Building on this, the study ran a two-stage phenotypic screen of 1232 repurposable compounds across familial non-SOD1 motor neuron models and then evaluated top hits in sALS lines. Ropinirole emerged as the top candidate, reducing apoptosis in a substantial subset of responsive sALS lines and improving readouts linked to oxidative stress and mitochondrial dysfunction, motivating subsequent clinical testing in the ROPALS trial [69].

Another major advance in ALS iPSC research has been the establishment of the Answer ALS (AALS) consortium [68]. This resource comprises patient-derived iPSC lines, multi-omic datasets from differentiated iPSC-MNs, and longitudinal clinical and smartphone-based data from more than 1000 ALS patients. Integration of these datasets using statistical modeling and AI is expected to identify disease-relevant biological signatures and to stratify patients into mechanistically distinct subgroups. A first characterization of iPSC-MNs derived from 92 control and 341 ALS Answer ALS iPSC lines, including sALS, revealed that cell composition and sex are significant sources of variability that need to be carefully controlled for in future studies [70]. In another Answer ALS consortium study profiling chromatin accessibility by ATAC-seq in iPSC-MN cultures from 380 ALS patients and 80 controls, much of the variance observed was explained by sex, cell-of-origin/reprogramming source, and technical sequencing factors [71]. Following adjustment, the most prominent ALS-linked signature was the reduced accessibility at the *C9orf72* promoter/TSS in *C9orf72* HRE lines. Importantly, machine learning models trained on ATAC-seq data predicted the rate of disease progression with accuracy comparable to methods based on blood biomarkers and clinical status [71].

Important insights into TDP-43 loss of function and nuclear pore complex injury in sALS derived from a study profiling iPSC-derived spinal neurons from 180 individuals (controls, *C9orf72* ALS/FTD, and sALS) obtained through the Answer ALS program [72]. Using a targeted qRT-PCR panel of 20 TDP-43-dependent transcripts/splicing events, the work found that ~86% of ALS lines showed at least one TDP-43 loss-of-function signature, including STMN2 or UNC13A misprocessing, with substantial variation in onset and severity across lines, and the same splicing abnormalities were also present in matched postmortem CNS tissue. These signatures were associated with nuclear pore pathology as indicated by nuclear CHMP7 accumulation, reduced POM121, and altered Ran distribution. POM121 depletion in controls induced TDP-43 loss-of-function readouts, whereas *CHMP7* ASOs or POM121 overexpression rescued splicing.

An independent large iPSC library from 100 sALS patients has been used for systematic phenotyping and pharmacological screening in iPSC-MNs [73]. These cultures showed robust reductions in neuronal survival, accelerated neurite degeneration, and transcriptional dysregulation that correlated with donor survival times. Responses to riluzole mirrored clinical benefit, and a retrospective screen of compounds previously tested in ALS trials revealed that 97% failed to rescue neurodegeneration in vitro, recapitulating the predominantly negative clinical outcomes. Importantly, forward combinatorial screen in the same system identified a triple regimen of baricitinib, memantine, and riluzole as a candidate therapeutic combination reaching 100% response in the highly heterogeneous sALS cohort. Collectively, these large-scale studies highlight the predictive validity of sALS iPSC models and their potential for patient stratification, responder prediction and drug discovery.

An important addition to the growing number of iPSC studies of sALS are the 3D and microphysiological iPSC models. A pioneering 3D motor-unit-on-chip combining optogenetic sALS iPSC-derived MNs with engineered 3D skeletal muscle showed reduced MN viability, weakened muscle contractions and NF-κB activation, with autophagy modulators (bosutinib, rapamycin) partially rescuing pathology, linking motor-unit failure to inflammatory and autophagy pathways in a human sALS background [39]. More recently, a perfused ALS organ-on-chip system with physically separated but functionally coupled MN and blood–brain barrier (BBB)-like endothelial compartments from sALS donors captured an early excitatory–inhibitory imbalance, with elevated glutamatergic signaling, reduced GABA receptor activity and more mature MN phenotypes than in 2D cultures [74]. This platform not only supports the idea that sALS involves early circuit-level hyperexcitability and synaptic dysregulation, but also brings in neurovascular and drug-penetration dimensions, enabling interrogation of how BBB properties and perfusion state influence MN vulnerability and therapeutic responses.

**Table 2:** Overview of key in vitro iPSC studies in sporadic ALS. Abbreviations: ↑, increased; ↓, reduced.

Study	Type of Study	iPSC Model	Differentiated Cell Type	Observed Phenotypes (sALS-Relevant)	Pharmacological Treatments
[61]	Patient iPSC disease modeling	Patient fibroblast-derived iPSCs (sALS + controls; cohort study)	iPSC-MNs	De novo TDP-43 aggregation in motor neurons from 3 sALS patients; aggregates recapitulated pathology seen in a matched postmortem sample	Digoxin as an example of TDP-43 aggregation modulator
[62]	Hallmark pathology modeling (familial + sporadic ALS)	Sendai virus–reprogrammed fibroblast iPSCs (2 sALS) + familial (*TARDBP* G298S); TALEN-edited H9 ESC AAVS locus model	iPSC-/ESC-MNs and non-MNs	TDP-43 aggregates in surviving MNs (fALS + sALS); ↑ neurofilament inclusions in ALS MNs; ↓ neurite mitochondrial density vs. controls; MNs show greater vulnerability under stress with apoptotic activation	MG132 (proteasome inhibitor) used as challenge → TDP-43 translocation, NF inclusions, impaired mitochondrial distribution, caspase-3 activation
[63]	Transcriptomics/gene expression profiling	sALS + control iPSC lines generated from motor nerve fibroblasts	iPSC-MNs	Gene expression dysregulation strongly associated with mitochondrial function and processes linked to motor neuron degeneration	-
[64]	Differentiation protocol + sALS phenotype readout	sALS + control iPSC lines	Cervical spinal motor neurons (csMNs)	Detection of hyperexcitability phenotypes in sALS iPSC-csMNs.	-
[66]	Early-mechanism study (axon biology)	sALS + control iPSC lines	iPSC-MNs, NMJ-related assays	Early phenotypes: impaired axonal transport, defective axonal outgrowth, reduced NMJ formation; transcriptomics implicate axon guidance pathway dysregulation including EphA4 and DCC upregulation	-
[67]	Population-scale iPSC disease modeling + phenotypic clustering + candidate therapy	Large panel of patient-derived iPSC models of sALS	iPSC-MNs (heterogeneity-focused)	Heterogeneous neuronal degeneration patterns, abnormal protein aggregate types, differing cell-death mechanisms, and variable onset/progression in vitro; case clustering framework across sALS models	Identified ropinirole as a multi-phenotype rescue candidate across subclassified sALS models
[68]	Large-scale resource/biobank + multi-omics + clinical linkage	Patient-derived iPSC lines (blood-derived), >1000 ALS participants with longitudinal data	iPSC-MNs + multi-omics (WGS, RNA, ATAC, proteomics) + clinical/smartphone data	Resource description (not a single-phenotype report), subtype discovery via integrated clinical–molecular signatures; open sharing portal	-
[70]	Large-scale differentiation/QC resource	341 ALS + 92 control iPSC lines from the Answer ALS consortium	iPSC-MNs	iPSC cohort characterization across 92 controls + 341 ALS motor neuron cultures; identified cell composition and sex as major sources of variability affecting downstream analyses	-
[71]	Epigenomics (ATAC-seq) at scale	380 ALS + 80 control iPSC lines from the Answer ALS consortium	iPSC-MNs	Chromatin accessibility by ATAC-seq strongly influenced by sex, iPSC origin, ancestry, sequencing variance ALS-specific signals post-correction ATAC features can predict disease progression rates comparably to biomarker/clinical methods	-
[72]	Population-scale iPSC neuron study of TDP-43 loss-of-function signatures	iPSC-derived neurons (iPSNs) from 180 individuals (controls, *C9orf72* ALS/FTD, and sALS)	iPSC-derived neurons (iPSNs); qRT-PCR panel + patient-matched postmortem validation	Identified variable, time-dependent molecular signatures of TDP-43 loss of function in iPSNs; same signatures seen in postmortem brain tissue from the same patients; linked nuclear pore integrity to TDP-43 dysfunction	POM121 reduction (nuclear pore injury) was sufficient to reproduce TDP-43-related molecular changes; repairing nuclear pore injury restored disrupted gene processing
[73]	Large-scale phenotypic screening + drug screening	iPSC library from 100 sALS patients	iPSC-MNs population-wide phenotypic screening	sALS MNs show reduced survival, accelerated neurite degeneration (correlating with donor survival), and transcriptional dysregulation; screen of prior ALS trial drugs shows 97% failed to mitigate neurodegeneration	Riluzole rescued neurodegeneration phenotypes; combinatorial testing identified baricitinib + memantine + riluzole as a promising combination
[39]	3D microphysiological ALS-on-a-chip motor unit	iPSC-derived optogenetic MN spheroids from a sALS patient + iPSC-derived skeletal muscle bundles	Microfluidic 3D NMJ model; optogenetic stimulation of MNs → muscle contraction readouts + iPSC-derived ECs (iECs) barrier	ALS motor unit shows fewer muscle contractions, MN degradation, and increased muscle apoptosis vs. non-ALS	Muscle contraction deficits improved with rapamycin and bosutinib (single and co-treatment); recovery associated with upregulated autophagy and TDP-43 degradation in MNs
[74]	Organ-on-chip (microfluidic) sALS model with BBB-like barrier	iPSC-MNs from early-onset sALS patients; iPSC-derived brain microvascular endothelial-like cells	Spinal cord chip (SC-chip) with flow + integrated BBB-like barrier	Flow improved maturation/health; transcriptomic/proteomic differences include increased neurofilaments; snRNA-seq identifies MN subpopulations and ALS-specific dysregulation of glutamatergic and synaptic signaling	-

## 5. From Dish to Clinic: Translating Findings to Effective Therapies in ALS

### 5.1. iPSC-Informed Clinical Trials in ALS

**Figure 3:**
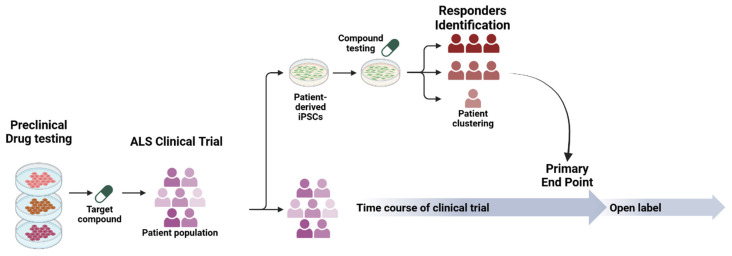
Integration of iPSCs into clinical trial pipelines. Compound testing in patient-derived iPSCs enables the prediction of drug responsiveness and patient stratification, generating translational insights that can be integrated with primary endpoint analyses in clinical trials. Created in BioRender. Michailidou, I. (2025) https://BioRender.com/1k2en1s (accessed on 21 January 2026).

The use of iPSC models holds promise in translational, patient-relevant therapeutic discovery and personalized clinical trials (Figure 3). Specifically, iPSC–MNs from ALS patients have been used as drug screening platforms to identify repurposed small molecules that can effectively rescue ALS phenotypes in vitro. Ezogabine (retigabine), ropinirole and bosutinib (Table 3) are examples of small molecules that showed efficacy in ALS iPSC-MN models and subsequently transitioned into ALS clinical trials in 2015, 2018 and 2019, respectively, establishing a clear bench-to-bedside paradigm in ALS research.

Ezogabine/retigabine, a Kv7 channel activator, was shown to normalize the hyperexcitability phenotype present across *SOD1*-, *C9orf72*- and *FUS*-mutant ALS iPSC-MNs and improve the survival of *SOD1*^A4V/+^ ALS iPSC-MNs [46]. These iPSC data, combined with clinical evidence of hyperexcitability in ALS and the lack of sALS animal models, provided the rationale for a phase 2 clinical trial testing whether ezogabine can reduce cortical and spinal motor neuron excitability in ALS patients [75]. The trial confirmed a reduction in motor neuron excitability in vivo, validating the clinical relevance of iPSC models and setting a paradigm for the use of neurophysiological metrics as pharmacodynamic biomarkers in clinical trials. However, despite its proof-of-mechanism in ALS, ezogabine was withdrawn from the global market in 2017 for reasons unrelated to ALS [76].

A high-throughput screen of 1232 FDA-approved drugs identified ropinirole, a dopamine D2 receptor agonist, as capable of rescuing ALS-like phenotypes, including neurite degeneration, oxidative stress and dysregulated lipid metabolism, in sALS iPSC-MNs [67]. These data led to the ROPALS phase 1/2a trial, which showed that ropinirole was well tolerated, and although the double-blind phase revealed only modest trends, longer-term open-label follow-up suggested slower functional decline and better preservation of daily activities in treated participants [69]. A unique feature of the ROPALS trial was the incorporation of reverse translational research whereby iPSC lines were generated from trial participants, and their iPSC-MNs were exposed to ropinirole in vitro. The extent to which ropinirole rescued cellular phenotypes, such as lipid peroxidation and mitochondrial dysfunction, paralleled the degree of clinical benefit in the corresponding patient, suggesting that iPSC-MN responses may serve as predictive or pharmacodynamic biomarkers. Furthermore, a longitudinal extracellular vesicle (EV) proteomics study embedded within the ROPALS cohort identified a shared sporadic ALS EV signature marked by increased inflammation/complement/coagulation proteins and reduced UPR/ER proteostasis proteins that correlated with functional status [55]. Baseline-to-week-24 comparisons suggested that ropinirole partially reversed these disease-associated EV changes, supporting EV cargo as a candidate biomarker and mechanism-linked pharmacodynamic readout. Reverse translational analyses using iPSC-astrocyte RNA-seq linked ropinirole to suppression of inflammatory transcriptional programs, providing a mechanistic explanation for the EV proteomic shifts observed in ROPALS EVs.

Similarly, bosutinib, an Src/c-Abl tyrosine kinase inhibitor, was prioritized via screening in ALS iPSC-MNs, where it reduced mislocalization and aggregation of TDP-43, reduced ER stress, and improved motor neuron survival [77]. This led to the iPSC-based Drug Repurposing for ALS Medicine (iDReAM) program, a phase 1 trial in Japan [78]. The trial demonstrated safety and tolerability across the tested dose range and provided exploratory evidence that bosutinib might slow ALSFRS-R decline and modify biomarker trajectories in some patients. A subsequent analysis of the extended iDReAM program reported no ALS-specific safety signals and suggested suppression of disease progression in a subset of treated participants, prompting planning of a larger phase 2 study. Similar to ropinirole, the iDReAM group has emphasized an integrated “bench-to-bedside-back” strategy, using iPSC-MNs and patient biomaterials to refine dose selection, mechanism of action, and potential stratification biomarkers.

Despite robust preclinical efficacy, two recent clinical trials of sense-strand ASOs (BIIB078, WVE-004) for *C9orf72*-ALS failed to demonstrate clinical benefit and raised concerns about worsening neurodegeneration, as evidenced by elevated neurofilament light chain (NfL) levels [79,80,81]. These trials were based on evidence from iPSC studies, among others, that ASOs targeting *C9orf72* repeat-containing sense transcripts can reduce hallmark molecular phenotypes such as RNA foci and downstream expression/splicing abnormalities [79,82].

**Table 3:** List of iPSC-informed clinical trials in ALS. Abbreviations: ASO, antisense oligonucleotide; sMNs, spinal motor neurons; HRE, hexanucleotide repeat expansion; DPR, dipeptide repeat protein.

Drug	Class	iPSC Model (Gene/Mutation)	Phenotype Rescue In Vitro	Clinical Trial and Trial ID	Reference
Ezogabine/Retigabine	Kv7 (KCNQ2/3) potassium-channel opener	iPSC-MNs from fALS (*SOD1* A4V × 2 lines, G85S, D90A, *C9orf72* HRE × 2 lines, and *FUS* M511FS, H517Q)	Normalized hyperexcitability (all genes), reduced ER stress markers (*SOD1*^A4V/+^), improved iPSC-MN survival (*SOD1*^A4V/+^)	NCT02450552 phase 2 ezogabine PD trial in ALS (completed)	[46,75]
Bosutinib	Src/c-Abl tyrosine kinase inhibitor	iPSC-MNs from fALS (*SOD1* H46R, *SOD1* L144FVX, *TARDBP* M337V) and sALS patients	Enhanced autophagy, reduced misfolded SOD1 and TDP-43 aggregates, restored mitochondrial homeostasis and improved iPSC-MN survival	NCT04744532 iDReAM phase 1/2 in ALS (ongoing)	[77,78]
Ropinirole	Dopamine D2/D3 receptor agonist	iPSC-sMNs from sALS patients	Rescued neurite retraction, autophagy defects, oxidative stress and cell death	UMIN000034954–ROPALS phase 1/2a (completed)	[67,69]
BIIB078 (IONIS-C9Rx)	ASO targeting *C9orf72* sense repeat-containing RNA	iPSC-MNs from *C9orf72* HRE ALS patients	Suppressed RNA foci, lowered DPRs and partially normalized gene expression	NCT03626012 phase 1 MAD safety/PK in C9-ALS (completed, no efficacy, program stopped); NCT04288856 open-label extension (terminated)	[79,80]
WVE-004	Stereopure ASO targeting repeat-containing *C9orf72* transcripts	iPSC-MNs and other neurons from *C9orf72* HREALS and FTD patients	Selectively reduced repeat-containing *C9orf72* transcripts, RNA foci and DPRs in C9 iPSC-MNs while sparing normal *C9orf72* protein	NCT04931862 FOCUS-C9 phase 1b/2a in C9-ALS/C9-FTD (terminated after lack of clinical benefit despite target engagement)	[81]

### 5.2. Challenges and Future Directions for Translational iPSC Models in ALS

Even in 2026, ALS treatment options remain limited. Riluzole and edaravone provide small average benefits, and tofersen, despite being a milestone, only applies to *SOD1*-ALS and it slows ALS progression rather than curing it. Meanwhile, clinical trial failures such as the negative phase 3 PHOENIX results and subsequent withdrawal of AMX0035/Relyvrio [83], or the termination of *C9orf72* ASO programs [80], keep highlighting the same uncomfortable lesson: successful preclinical interventions do not always translate to clinical benefit for patients. iPSC-based ALS models are meant to narrow the translational gap by providing human, genetically defined platforms for mechanism discovery and drug testing. But why do they occasionally fail to predict clinical efficacy? One major pitfall is targeting the wrong mechanism. A reproducible iPSC phenotype is necessary, but it is not sufficient to establish a clinically actionable disease driver. A phenotype becomes clinically meaningful when it is mechanistically upstream rather than a downstream consequence or compensatory response, shows mechanistic specificity, aligns with patient biology and biomarkers, and when its modulation yields durable functional protection. Even when the mechanism is valid and relevant in vivo, the context may be missing as certain phenotypes depend on multicellular interactions, immune contributions, or circuit-level dynamics that are difficult to capture in reductionist iPSC systems and require complex NMJ, organoid and 3D organ-on-chip models. Additional factors can also drive failure in iPSC-informed trials, including mismatches in intervention timing, as well as insufficient drug dosing and CNS distribution. Importantly, weak or negative phenotypes across subsets of sporadic ALS (sALS) iPSC lines should not automatically be dismissed as noise. They may instead reflect genuine biological heterogeneity and can inform responder/non-responder stratification based on genotype or molecular signatures. Finally, iPSC evidence should support clinical progression not on the basis of statistically significant phenotypic rescue alone, but on robust, reproducible across multiple lines and sites, and most importantly linked to neurodegeneration-relevant endpoints and human biomarkers. In this section, we discuss further the challenges that need to be addressed so iPSC platforms can be used confidently to guide prioritization of therapeutic candidates for drug development pipelines [84].

One important issue is that iPSC reprogramming largely resets cellular age [85]. This is a major challenge for modeling late-onset neurodegenerative diseases such as ALS. In this context, genetic predisposition is the “first hit” and aging is the “second hit” for ALS disease development. In vitro iPSC-derived models are young and immature compared to the stressed and aged neurons of ALS patients [86], which can influence the detection and translatability of in vitro phenotypes. These iPSC systems may be viewed as equivalents of pre-symptomatic modeling rather than end-stage, where genetic information drives some of the earliest detected phenotypes that over time and with the contribution of aging-related stress can lead to overt neurodegeneration. As the lack of aging signatures is a widely acknowledged limitation of the iPSC technology, several methods to induce aging phenotypes have been described, including direct conversion of fibroblasts to neurons (induced neurons, iNs) [87], induction of aging via progerin overexpression [88], telomere shortening [89], or even longer in vitro maturation. Each of these methodologies offers potential advantages but also distinct trade-offs. For instance, iNs may better retain donor age-associated transcriptomic signatures [86], but are less scalable and associated with batch-to-batch variability issues. Progerin-induced accelerated aging can help reveal age-related phenotypes in vitro but may introduce non-physiological stress responses [88]. Telomere shortening induces aging-related features, such as DNA damage, mitochondrial ROS generation, and dendritic atrophy in iPSC-derived neurons; however, the telomere length is variable and the impact of telomere shortening on post-mitotic cells remains to be studied [89]. Finally, long-term maturation can improve functional maturity of cultured iPSC-MNs but is tied to increased time and cost, and is prone to culture drift and selective pressure favoring specific cell types. Despite addressing a key limitation, these methods are not yet routine or standardized, which currently limits their translational value. Nevertheless, induced aging can be used as an additional “second-hit” parameter to address whether candidate phenotypes observed in “young” iPSC-MN models persist or intensify, thereby strengthening their potential clinical relevance.

Another major hurdle is the variability affecting the robustness and reproducibility of iPSC phenotypes. Identifying and eliminating all sources of variability, where possible, are necessary steps to increase robustness of the iPSC models. Discrepancies in the reported phenotypes are a very common finding across different ALS iPSC studies that complicate the interpretation of the results. This is largely due to all the variation associated with different experimental parameters, including different differentiation protocols, efficiencies, cellular composition, culture duration and stress, among many more. Recent large-scale studies show that covariates not directly related to disease biology, such as cell-type composition and batch, can dominate transcriptomic and epigenomic signals if they are not explicitly measured and modeled [70]. Even though variability is difficult to eliminate entirely, it can be substantially reduced and better controlled through protocol standardization, the use of shared reference metrics and materials, and harmonized quality-control benchmarks across sites. This is a key reason why consortia such as Answer ALS have emerged in order to generate familial and sporadic iPSCs at scale under harmonized conditions, pair them with deep molecular and clinical annotation, and make the resulting datasets broadly available.

Apart from experiment-driven variability, iPSC models also exhibit inherent variability arising from the heterogeneous clinicopathogenetic landscape of ALS, in which distinct genetic architectures, molecular endotypes, and clinical manifestations can produce divergent cellular phenotypes even under standardized differentiation conditions. This becomes obvious when many patient iPSC-derived lines are tested side-by-side, with some lines showing strong degeneration or aggregation phenotypes, whilst others display subtle or different signatures under identical conditions. That variability is a major obstacle when the goal is a single, universal screening endpoint, but it also provides a strong rationale for mechanism-based stratification and subgroup-specific therapeutic development. Importantly, heterogeneity becomes tractable when datasets get large enough. For example, Answer ALS cohort ATAC-seq in iPSC-MNs suggests that chromatin accessibility patterns can separate ALS from controls and even predict progression rates [71]. This suggests that ALS drug development may need to increasingly focus on therapies evaluated in biologically defined patient subsets enriched for the targetable mechanism.

Even if an in vitro phenotype is robust and reproducible, the big question of whether it is clinically relevant and translational remains. Many iPSC studies rely on measurable and scalable endpoints such as neurite outgrowth, resistance to an acute stressor, and mitochondrial potential. Clinical trials, however, are judged on ALSFRS-R decline, survival, ventilation milestones, and respiratory function over many months. There is no universally accepted shared endpoint. Recent large iPSC studies, however, provide hope. In a large sALS iPSC-MN screening study, riluzole produced modest rescue consistent with its limited clinical benefit, and most compounds with prior clinical failure did not rescue the in vitro degeneration phenotype [73].

**Figure 4:**
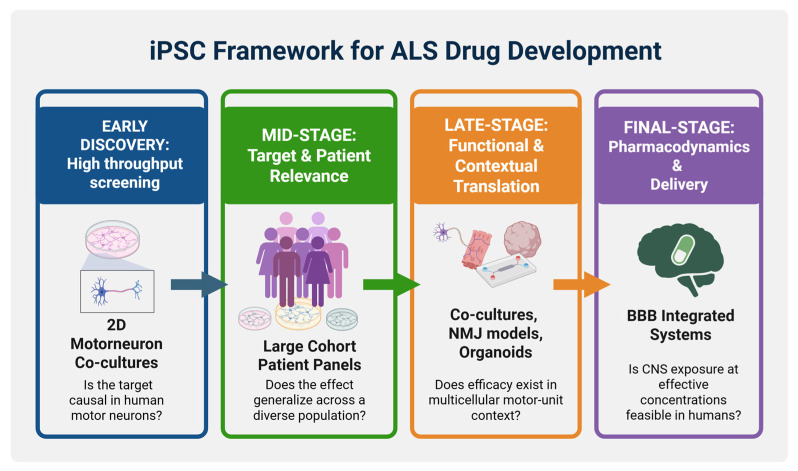
Proposed iPSC framework for translational ALS drug discovery. Different iPSC models can be used during different stages of ALS drug development, based on the specific translational questions at each stage. Early discovery prioritizes 2D iPSC-derived motor neuron cultures for high-throughput screening, dose–response relationships, and low-variability phenotypic readouts to address the questions: “Is the pathway causal in human motor neurons?” and “Is there a tractable cellular phenotype?”. Once a candidate drug and readout are established, large-cohort sALS iPSC platforms are prioritized to quantify heterogeneity and determine whether the phenotype and drug response are consistent across genetically diverse patients, or restricted to specific subgroups, supporting responder identification, molecular signatures, biomarker selection, and patient stratification for clinical trials. In late-stage discovery, more complex, lower-throughput systems (NMJ models, 3D organoids) test motor-unit function, circuit-relevant phenotypes, and non-cell-autonomous mechanisms to address whether efficacy persists when these contextual features are represented. In parallel, organ-on-chip and BBB-integrated platforms evaluate pharmacodynamics and delivery by asking “Can the drug reach relevant CNS compartments at effective concentrations, and does it engage the target under realistic exposure conditions?”. Created in BioRender. Michailidou, I. (2026) https://BioRender.com/kdbs0jt (accessed on 21 January 2026).

Another practical translation gap is that typical 2D cultures and many 3D systems do not model drug exposure constraints or important physiology such as neuromuscular connectivity, muscle contraction, BBB permeability, peripheral immune interactions, and muscle-to-neuron feedback. Microphysiological systems such as organ-on-chip, BBB-integrated platforms and NMJ-on-chip are promising here because they offer a physiologically relevant system to test pharmacology and circuit-relevant phenotypes. However, due to their expensive and technically demanding nature, they have limited use in routine drug screening. In the near future, these complex human microphysiological systems are likely to be used for higher-confidence secondary validation of candidate drugs emerging from 2D primary screens, thereby strengthening the evidence base for candidate prioritization in clinical trial (Figure 4).

The recent failure of *C9orf72* ASO trials highlights more issues with translatability of robust and reproducible target engagement in iPSC-MNs [80]. *C9orf72* ASO efforts, including BIIB078, showed evidence of molecular engagement but did not translate to clear benefit. Future strategies should go beyond sense RNA targeting, as antisense RNA has been shown to sufficiently drive TDP-43 pathology [90] in recent iPSC studies [90,91]. Other important parameters to consider include correction of downstream proteostasis and excitability defects, and preservation of C9orf72 function. Such outcomes argue for a stricter standard in human models whereby candidates should demonstrate downstream correction of convergent disease biology not only upstream target modulation, and those corrections should be tied to biomarkers that can be measured in patients.

A further translational bottleneck is the incomplete integration of shared biomarkers across the bench-to-bedside loop. In the absence of a common biomarker, iPSC phenotypes often struggle to inform practical clinical questions such as dose selection, expected time-to-effect, or early futility criteria. Neurofilament light chain (NfL) is beginning to play this unifying role in ALS drug development, with growing consensus around its utility as a prognostic and response biomarker. The regulatory relevance of NfL was highlighted by the accelerated-approval pathway for tofersen in *SOD1*-ALS, where reductions in plasma NfL were used as a reasonably likely surrogate endpoint. In parallel, the ATLAS study in clinically presymptomatic *SOD1* variant carriers uses elevated plasma NfL as biomarker evidence of disease activity to support initiation and assessment of the treatment [92].

Timing of intervention is another issue impacting translatability of iPSC models. In vitro iPSC treatments rescue some of the earliest, likely pre-symptomatic phenotypes whereas many ALS trials are conducted in patients already exhibiting irreversible motor neuron loss, advanced neuroinflammation, and systemic decline. This temporal mismatch coupled with the irreversibility of established neurodegeneration significantly reduces therapeutic benefit even for a mechanistically sound therapy. Therefore, the ALS field is increasingly moving towards earlier, genetics- and biomarker-enabled study designs. This is also reflected by the ATLAS study that aims to administer tofersen in clinically presymptomatic *SOD1* variant carriers upon biomarker evidence, including elevated plasma NfL, with the goal of delaying phenoconversion [92].

Considering the heterogeneity of ALS, single compounds may not be the only option for therapy. Translational iPSC platforms have not only identified single compounds but have also pointed towards drug combination strategies that are supported across diverse donor lines and mechanistically complementary rather than additive. This is highlighted by a large-scale screening of sALS iPSC-MNs, which has demonstrated that most previously trialed compounds failed to rescue survival across ALS iPSC-MNs, riluzole showed only a modest effect consistent with its limited clinical benefit [73]. In contrast, two-drug or three-drug combinations of the effective agents, baricitinib, memantine, and riluzole resulted in strong rescue in ~87% and 100% of the lines, respectively, demonstrating that drug combinations can broaden and deepen response across heterogeneous sALS donors [73]. Importantly, as several ALS iPSC studies have highlighted a significant contribution of neuroinflammatory processes in ALS pathogenicity, considering combination therapies that include anti-inflammatory agents may be of use in ALS [93,94]. In line with this, in a 3D *C9orf72* spinal microtissue (SM) model, consisting of iPSC-MNs, iPSC-astrocytes and iPSC-microglia, telmisartan treatment could protect iPSC-MNs against *C9orf72*-microglia-mediated toxicity by reducing levels of secreted interleukin-6 (IL-6) and IL-8, highlighting the importance of anti-inflammatory strategies and their therapeutic value in ALS [95].

In conclusion, iPSC models hold great translational value which can be significantly strengthened by the use of a decision-oriented framework guiding the selection of different iPSC platforms based on the specific translational questions during the drug discovery process (Figure 4). At the early discovery stage, 2D iPSC-derived motor neuron cultures are the most suitable platforms as they offer high-throughput screening based on well-defined phenotypic readouts and dose–response relationships with reduced variability and cost. Once a candidate drug and readout are established, the next important stage in the drug development process is to address the target relevance across heterogeneous ALS backgrounds. For this purpose, large-cohort sALS iPSC platforms can be prioritized to quantify heterogeneity, identify responders, and molecular signatures that can support patient stratification and biomarker selection for subsequent clinical trials. During the last stages of drug discovery, the selective use of more complex and low-throughput iPSC platforms such as NMJ models and 3D organoids are important for testing motor-unit function, non-cell-autonomous mechanisms, and circuit-relevant phenotypes. In parallel, organ-on-chip and BBB-integrated platforms are most valuable for addressing pharmacodynamics and drug delivery by evaluating target engagement and efficacy under more realistic conditions.

## 6. Concluding Remarks

Early diagnosis, the development of reliable biomarkers, and stratification of patients based on genetic and phenotypic subtypes may enable precision medicine approaches in ALS management. From a disease modeling perspective, iPSCs have transformed ALS research and the wider neurodegeneration field by allowing human disease mechanisms to be studied in a robust, patient-specific and physiologically relevant manner. Viewed across the timeline of ALS iPSC modeling evolution, steady methodological gains have moved the field from largely motor neuron-centric to deeper interrogation of non-cell-autonomous glial influences and, more recently, to multicellular 3D motor-unit platforms that better approximate key aspects of human neuromuscular physiology. These advances have improved translatability potential and expanded what can be asked, linking molecular pathology to circuit or NMJ-level dysfunction in human-relevant contexts for mechanistic discovery and drug screening. Yet this experimental progress has not translated proportionally into therapeutic impact, as ALS treatments remain limited and clinically modest in effect. Addressing this gap will likely require incorporation of translational iPSC frameworks into the drug development programs and tighter coupling of iPSC platforms to patient data through reverse translation research paradigms.

## Data Availability

No new data were created or analyzed in this study.
